# The emotional brainbow

**Published:** 2019-07-24

**Authors:** Luckshi Rajendran

**Affiliations:** 1 Faculty of Medicine, University of British Columbia

**Figure:**
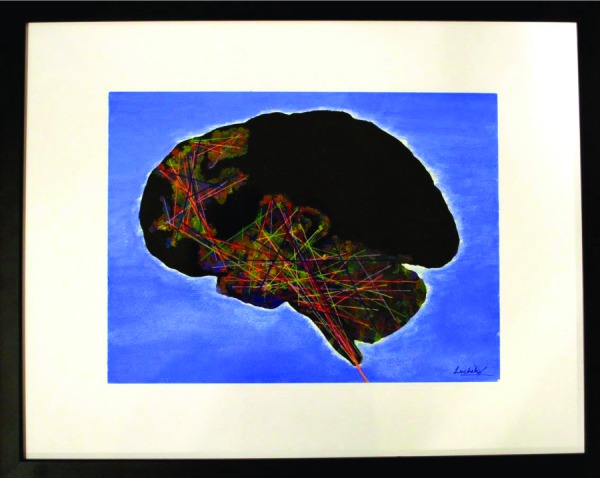


It was early in my first year of medical school that I learned about the “brainbow” - an innovative means of using genetic expression of various fluorescent proteins to colourfully label individual neurons, allowing for the visualization of neural networks within the brain. I was fascinated by the beautiful complexity of these axonal interconnections. In reflection, I drew parallels to my journey through medicine, and the intricacies of navigating human interpersonal relationships.

Medical practice includes both the soft and the hard sciences. Academic institutions teach us the hard sciences: the pathophysiology of disease, and the evidence-based practice for diagnosis and management. Over the years of my clinical training, I am learning that much of the soft science of medicine is in the human connection. It is in our ongoing practice of communication and interpersonal skills, and the subsequent relationships that we develop (or sometimes, lose) with our friends, partners, and colleagues, as we face the miracles and the hardships throughout our medical training. It is in our patient interactions: the emotions we share, the empathy we convey, and the rapport that we build in order to provide compassionate patient care. Much like the brain’s neural network, these connections are complex and ever-changing - some connections are strengthened, and others are unfortunately, and perhaps painfully, pruned.

My piece “The emotional brainbow” uses fine multicolours of sewn thread to reflect the intricate axonal connections of brain centres involved in processing and expressing emotions: the cortex, the limbic system, the brainstem, and the cerebellum. These crucial structures communicate to facilitate our ability to understand and empathize with others, and contributes towards our continually developing practice of manoeuvering interpersonal relationships. There is a complex, overlapping interplay of these neural connections within the emotion-regulating brain centres, much like the beautifully intricate emotional human connections, which we, as health care professionals, both create and navigate.

## About the author

Luckshi Rajendran is a fourth-year medical student at the University of British Columbia, Vancouver-Fraser Medical Program, and a soon-to-be General Surgery resident at the University of Toronto. Collectively, the arts have played a significant role throughout her medical training and personal development, as a mental escape, an outlet for self-expression, and a tool for medical education. She is especially grateful to the arts for the opportunity to build strong human connections with many inspirational mentors and role-models over the last four years. Outside of the arts and medicine, she greatly enjoys being active and outdoors, particularly running and hiking, and exploring new things.

